# Evaluation of two highly effective lipid-lowering therapies in subjects with acute myocardial infarction

**DOI:** 10.1038/s41598-021-95455-z

**Published:** 2021-08-05

**Authors:** Aline Klassen, Andrea Tedesco Faccio, Carolina Raissa Costa Picossi, Priscilla Bento Matos Cruz Derogis, Carlos Eduardo dos Santos Ferreira, Aline Soriano Lopes, Alessandra Sussulini, Elisa Castañeda Santa Cruz, Rafaela Tudela Bastos, Stefanie Caroline Fontoura, Antonio Martins Figueiredo Neto, Marina Franco Maggi Tavares, Maria Cristina Izar, Francisco Antonio Helfenstein Fonseca

**Affiliations:** 1 Department of Chemistry, Federal University of Sao Paulo (UNIFESP), Diadema, SP Brazil; 2 Center for Multiplatform Metabolomics Studies (CEMM), Institute of Chemistry, University of Sao Paulo (USP), Sao Paulo, SP Brazil; 3 Hospital Israelita Albert Einstein, Sao Paulo, SP Brazil; 4 Department of Analytical Chemistry, Laboratory of Bioanalytics and Integrated Omics (LaBIOmics), Institute of Chemistry, University of Campinas (UNICAMP), P.O. Box 6154, Campinas, SP 13083-970 Brazil; 5 Institute of Physics, University of Sao Paulo (USP), Sao Paulo, SP Brazil; 6 Division of Cardiology, Department of Medicine, Federal University of Sao Paulo (UNIFESP), Rua Loefgren 1350, São Paulo, SP CEP 04040-001 Brazil

**Keywords:** Lipidomics, Fatty acids

## Abstract

For cardiovascular disease prevention, statins alone or combined with ezetimibe have been recommended to achieve low-density lipoprotein cholesterol targets, but their effects on other lipids are less reported. This study was designed to examine lipid changes in subjects with ST-segment elevation myocardial infarction (STEMI) after two highly effective lipid-lowering therapies. Twenty patients with STEMI were randomized to be treated with rosuvastatin 20 mg QD or simvastatin 40 mg combined with ezetimibe 10 mg QD for 30 days. Fasting blood samples were collected on the first day (D1) and after 30 days (D30). Lipidomic analysis was performed using the Lipidyzer platform. Similar classic lipid profile was obtained in both groups of lipid-lowering therapies. However, differences with the lipidomic analysis were observed between D30 and D1 for most of the analyzed classes. Differences were noted with lipid-lowering therapies for lipids such as FA, LPC, PC, PE, CE, Cer, and SM, notably in patients treated with rosuvastatin. Correlation studies between classic lipid profiles and lipidomic results showed different information. These findings seem relevant, due to the involvement of these lipid classes in crucial mechanisms of atherosclerosis, and may account for residual cardiovascular risk.

Randomized clinical trial: ClinicalTrials.gov, NCT02428374, registered on 28/09/2014.

## Introduction

Therapies aiming to reduce LDL-C changed substantially the natural history of cardiovascular disease (CVD), especially coronary heart disease (CHD)^1^. However, despite the achievement of very low levels of LDL-C, recurrent events after acute coronary syndromes are still observed, suggesting that other lipid components including fatty acids (FA) may contribute to the atherothrombotic disease^2–4^. In addition, decreased plasma levels of plasmalogens were reported in subjects with CHD^5^. In fact, their role in atherosclerosis is still poorly understood, involving possibly antioxidant properties that impact the movement of molecules in and out of the cells, as plasmalogens are components of cell membranes^6^. High free FA concentrations can induce activation of NLRP3 inflammasome, triggering a pro-inflammatory response related to atherosclerosis, and lipid-lowering therapies, such as statin combined with the inhibitor of intestinal cholesterol absorption (simvastatin + ezetimibe), can partially revert many inflammatory biomarkers^7–9^. Following rosuvastatin treatment, a lipidomic study revealed significant decrease in sphingomyelin (SM), triglycerides (TG), phosphatidylinositol (PI) and phosphatidylethanolamines (PE) levels, but for lysophosphatidylcholines (LPC) and phosphatidylcholines (PC), no significant changes were reported^10^. Despite effective achievement of LDL-C and non HDL-C targets by the use of less potent statin combined with ezetimibe, their effects in other lipids are less reported. Therefore, this study aimed to compare lipid composition in the plasma of subjects with very high cardiovascular risk with STEMI at baseline (first day) and after 30 days of exposure to two highly effective lipid-lowering therapies (rosuvastatin alone or simvastatin combined with ezetimibe).

## Results

### Classic lipid parameters

**Figure 1:**
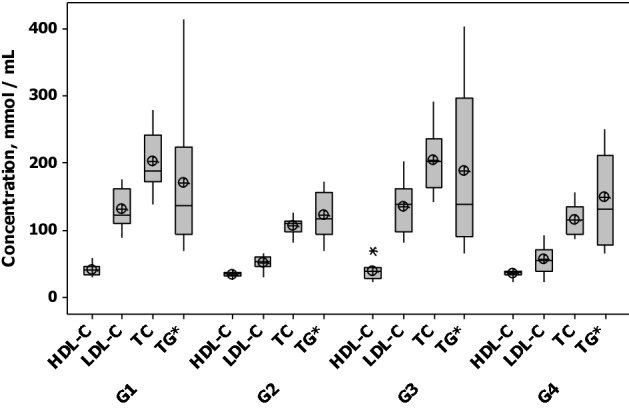
Evaluation of clinical results for the infarcted patients under investigation. G1: Patients randomized to the rosuvastatin group at the first day of myocardial infarction (D1); G2: Patients treated by rosuvastatin after 30 days (D30); G3: Patients randomized to the simvastatin + ezetimibe group at the first day of myocardial infarction (D1); G4: Patients treated by simvastatin plus ezetimibe after 30 days (D30). Total cholesterol (TC), low density lipoprotein—cholesterol (LDL-C), high density lipoprotein—cholesterol (HDL-C) and triglycerides (TG*). Figure was created in Minitab 17.0 (Minitab Statistical Software; URL: https://www.minitab.com/pt-br/)) and Microsoft PowerPoint 2013 (URL: https://www.microsoft.com/pt-br/microsoft-powerpoint-2013).

Prior to the lipidomic study, a classic lipid profile was obtained. Figure 1 shows the box plots of measurements of total cholesterol (TC), high-density lipoprotein-cholesterol (HDL-C), low-density lipoprotein cholesterol (LDL-C) and triglycerides (TG*) in each group of samples. As expected, after one month of both statin therapies, main changes were observed for TC (q < 0.001) and LDL-C (q < 0.001) with comparable magnitude for both treatments as observed by similar % changes and *q*-values from Post Hoc test with Bonferroni correction for multiple comparisons of the repeated measures ANOVA (Table S8). It was observed that rosuvastatin therapy decreased on a small scale HDL-C at D30 (− 17%, *q-*value = 0.04).

### Lipid class evaluation

**Figure 2:**
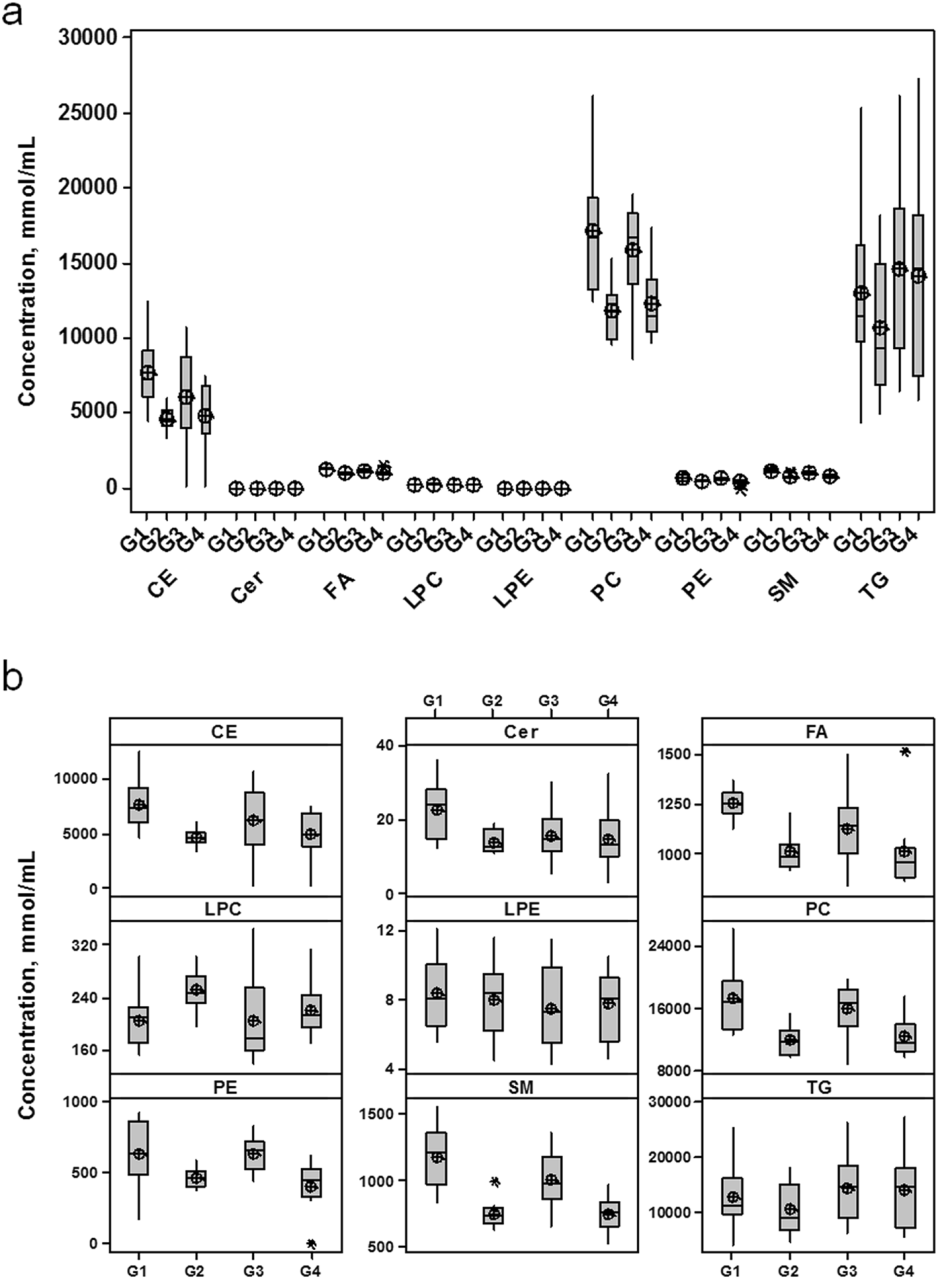
Evaluation of lipid classes for the infarcted patients under evaluation itemized by class (**a**) and by group (**b**). *CE* cholesterol ester, *Cer* ceramides, *FA* free fat acids, *LPC* lysophosphatidylcholine, *LPE* lysophosphatidylethanolamine, *PC* phosphatidyl choline, *PE* phosphatidylethanolamine, *SM* sphingomyelin, *TG* triacylglycerides; group labels as in Fig. 1. Figure was created in Minitab 17.0 (Minitab Statistical Software; URL: https://www.minitab.com/pt-br/) and Microsoft PowerPoint 2013 (URL: https://www.microsoft.com/pt-br/microsoft-powerpoint-2013). ±Mean Value.

Figure 2 shows the plasma concentrations of the analyzed lipids, categorized by classes (CE, Cer, FA, LPC, LPE, PC, PE, SM, and TG) (Fig. 2a) and by groups (G1, G2, G3 and G4) (Fig. 2b). Comparing the mean values in each group, lipid classes were decreased in both groups after the exposure to the treatments at D30, except for LPC, LPE and TG compound class. However, significant changes were observed for FA, LPC, PC, PE, SM, CE, and Cer only for rosuvastatin group, while SM changed in both groups, as evaluated by *q*-values from post hoc test with Bonferroni correction for multiple comparisons of the repeated measures ANOVA (Table S8).

Considering rosuvastatin administration at D30, there was a decrease for CE (− 40%, *q*-value = 0.006), Cer (− 37%, *q*-value = 0.02), FA (− 19%, *q*-value = 0.006), PC (− 65%, *q*-value < 0.001), PE (− 63%, *q*-value < 0.001) and SM (− 36%, q < 0.001). LPC was the only lipid class that increased (27%, *q*-value = 0.002) in G2. Following simvastatin plus ezetimibe therapy at D30, there was a decrease in SM (− 27%, *q*-value = 0.002). Although not significant (*q*-value > 0.05), simvastatin treatment after D30 also showed a trend for decrease in PE (− 36%, *q*-value = 0.08) and PC (− 23%, *q*-value = 0.1). FA, CE, Cer, LPC, TG and LPE were almost unaltered in G4 (*q*-value > 0.05, % change: between − 11% and 11%). Comparing the two arms of treatment at D1 (G1 and G3) lower levels of PC (− 54%, *q*-value < 0.001) and PE (− 51%, *q*-value = 0.001) were found for simvastatin plus ezetimibe group. Regarding the effects of treatments at D30 (G2 and G4), only the sum of LPC were 14% decreased (*q*-value = 0.02) in G4 compared to rosuvastatin arm (G2).

### Correlation studies

**Figure 3:**
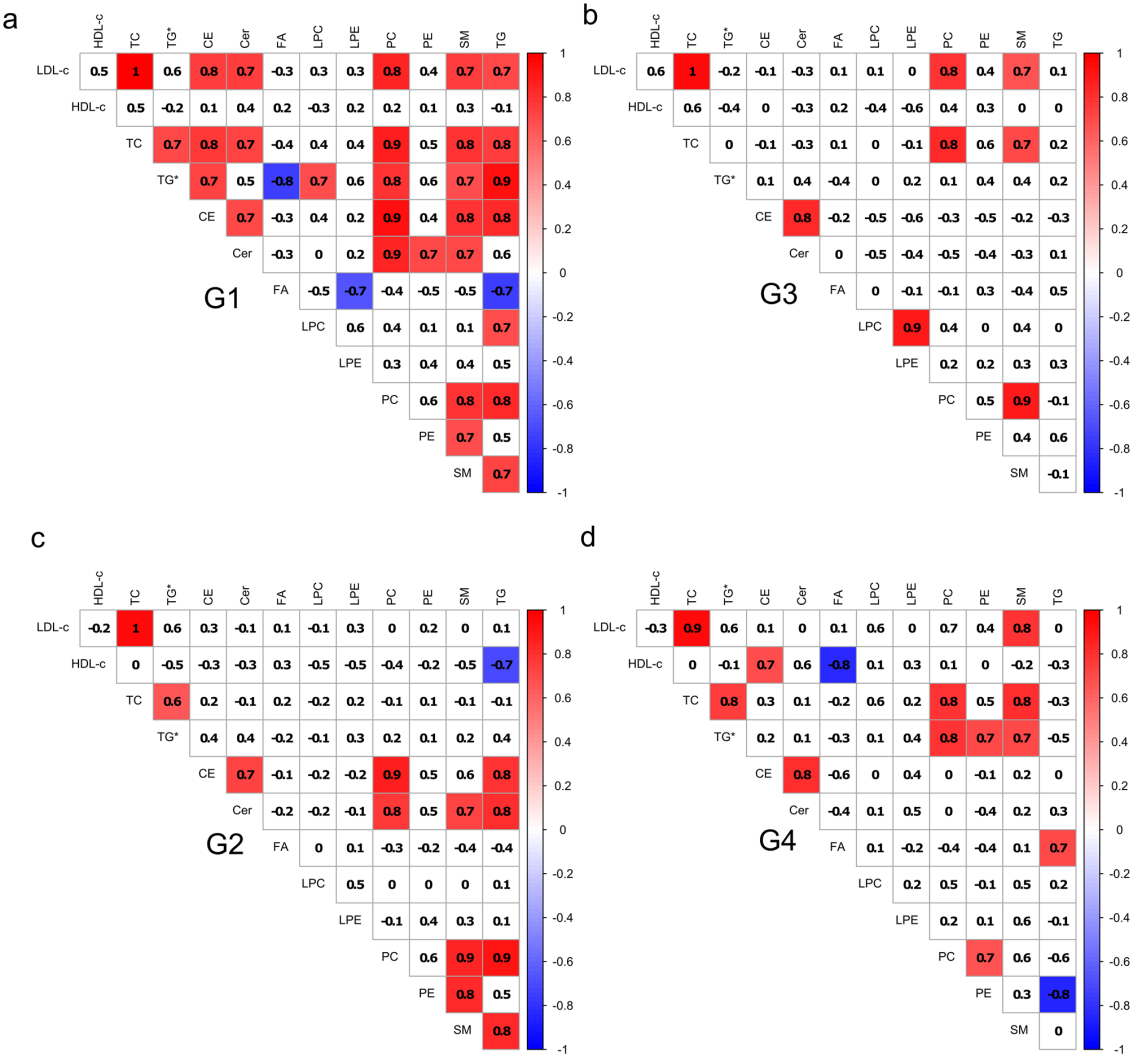
Pearson’s correlation of lipid classes concentrations and clinical parameters. (**a**) for rosuvastatin treatment at D1 (G1), (**b**) for simvastatin plus ezetimibe treatment at D1 (G3), (**c**) for rosuvastatin treatment (G2) at D30, and (**d**) for simvastatin plus ezetimibe treatment at D30 (G4). Figure was created in R 3.6.3 (The R Project for Statistical Computing; https://www.r-project.org/packages: corrplot, Hmisc, RColorBrewer, Cairo).

Levels of LDL-C, HDL-C, TC, and TG* were correlated with levels of lipid classes (CE, Cer, FA, LPC, LPE, PC, PE, SM, and TG) for all studied patient groups (Fig. 3). More correlations between clinical parameters and lipids were found considering rosuvastatin administration at D1 in comparison to D30 and to the other lipid-lowering therapy. Figure 3a,b present the correlation plots related to the two therapies at D1 of STEMI, respectively, to G1 and G3. Considering rosuvastatin administration at D1 (Fig. 3a) a series of correlations between clinical parameters and lipid classes were observed. CE, PC, SM and TG were highly correlated with LDL-C, TC and TG*. Otherwise, TG* was negatively correlated to FA. Interestingly, considering simvastatin plus ezetimibe at D1 (Fig. 3b), no negative correlation was found and fewer positive correlations are present. PC was positively correlated to LDL-C and TC, and SM was positively correlated only with TC. Figure 3c,d depicts correlation plots related to the two therapies at D30 of STEMI, respectively, to G2 and G4. Considering rosuvastatin at D30 (G2), only one negative correlation between TG and HDL-C was found. Positive correlations between PC vs. LDL-C and TG*; SM vs. LDL-C and TC; LPC vs. LDL-C were found considering simvastatin plus ezetimibe at D30 and, a negative correlation was found between FA and HDL-C.

### Identification of statistically significant lipids

#### Univariate and multivariate analyses

**Figure 4:**
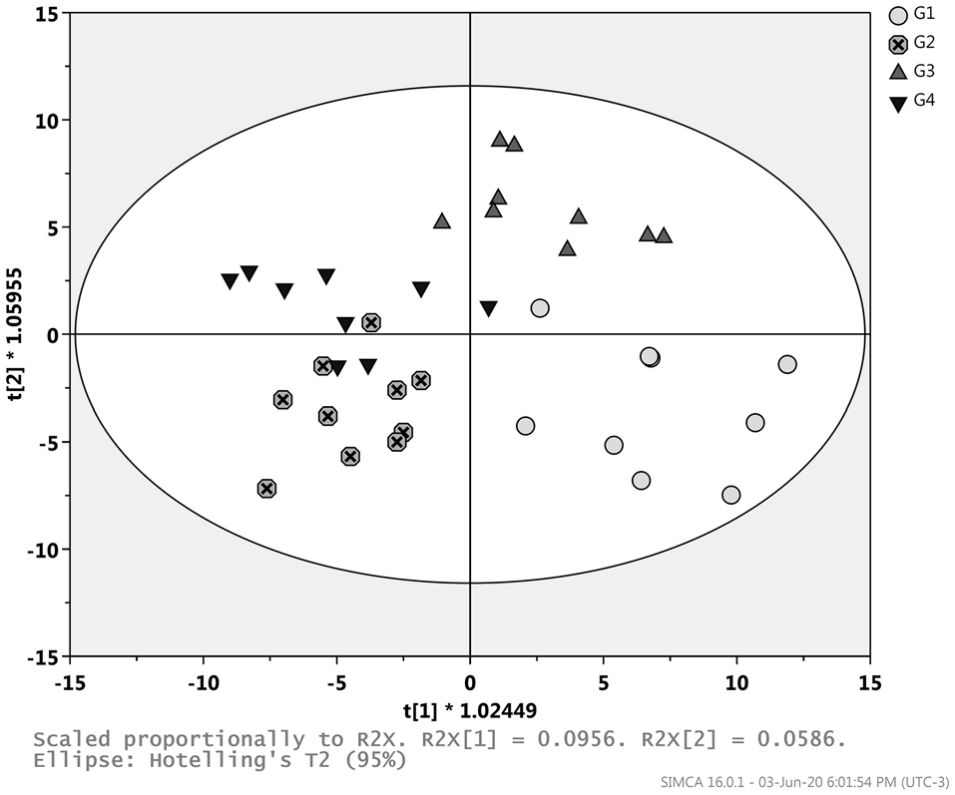
OPLS-DA scores plot (PC1 vs PC2) of all infarcted patients in the four groups considered. G1: Patients randomized to the rosuvastatin group at first day after myocardial infarction (D1); G2: Patients treated by rosuvastatin after 30 days of treatment (D30); G3: Patients randomized to the simvastatin plus ezetimibe group at the first day after myocardial infarction (D1); G4: Patients treated by simvastatin plus ezetimibe after 30 days (D30). Figure was created in SIMCA 16 (Statistical Software Package, Umetrics, Sweden; URL: http://umetrics.com/product/simca).

The OPLS-DA scores plot (Fig. 4) shows a separation among the four studied groups (G1, G2, G3 and G4). It is possible to observe a higher separation between time of treatment, D1 (G1 and G3) and D30 (G2 and G4), in comparison to the separation between the two lipid-lowering therapies, rosuvastatin (G1 and G2) and simvastatin plus ezetimibe (G3 and G4). However, a worst separation was observed between the two lipid-lowering therapies at D30 (G2 × G4). The OPLS-DA model, with R^2^ = 0.637 and Q^2^ = 0.246, was validated by CV-ANOVA (*p*-value = 0.032) and permutation test (100 permutations) of selected Y variables. The poor predictability of the model, indicated by Q^2^ < 0.5, is provided by the low numbers of observations (n = 10) and the properties of the dataset. Thus, this model must be validated by permutation test and by cross-validation (*p*_cv-ANOVA_)^11^. The Q^2^VY values for each y variables, from the permutation test, had shown that the Y variable G4 was responsible for the poor predictability of this model (Q^2^ = 0.246) as shown in the Supplementary Table S1. Triba et al., recommend to permute the lines of their dataset to control that the Q^2^ value calculated is stable regarding this permutation^11^. In addition, the stable Q^2^ with low error (0.251 ± 0.06; Q2 mean ± 2*Error; Error = t_a2_*sd/√n) obtained towards a permutation test with five randomized dataset, indicating that the model is trustful (See Supplementary Table S2).

**Figure 5:**
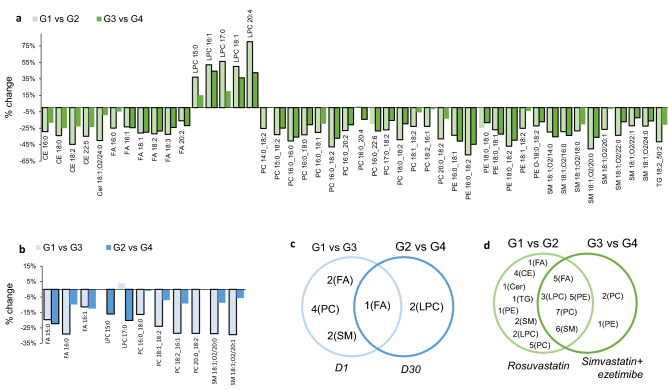
Discriminant lipids from multivariate (VIP > 1) and univariate analysis (repeated measures ANOVA). (**a**) % change of metabolites considering the temporal treatment effect (*metabolite statistically significant are highlighted with black borders); (**b**) % of change of metabolites considering the lipid-lowering therapy effect (*metabolite statistically significant are highlighted with black border); (**c**) Venn Diagram considering lipid-lowering therapy; (**d**) Venn Diagram considering temporal treatment effect. Figure was created in Microsoft Excel 2013 (URL: https://www.microsoft.com/pt-br/microsoft-excel-2013) and Microsoft PowerPoint 2013 (URL: https://www.microsoft.com/pt-br/microsoft-powerpoint-2013).

Discriminant variables were selected by the combination of multivariate analysis (VIP > 1), and univariate evaluation by post hoc test with Bonferroni correction for multiple comparisons of the repeated measures ANOVA (q-value < 0.05 for one of the comparisons) as shown in Supplementary Table S3 and displayed in Fig. 5. VIP value for all variables studied and, post-hoc (*q-value*) for variables considered statistically significant are shown in Supplementary Table S4 and S5, respectively.

Figure 5a,b show the percentage change related to the two lipid-lowering therapy effect (G1 vs G3 and G2 vs G4) and temporal treatment effect (G1 vs G2 and G3 vs G4). As expected, fewer metabolites are altered related to the two lipid-lowering therapy comparison than temporal treatment. Figure 5c shows that 2 FA, 4 PC and 2 SM were significantly altered between the two lipid-lowering therapies at D1 (G1 vs G3), only 2 LPC at D30 (G2 vs G4) and, FA 16:1 was similar for both comparison (D1 and D30).

Considering the temporal treatment evaluation (G1 vs G2 and G3 vs G4), Fig. 5d shows that 1 FA, 4 CE, 1 Cer, 1 TG, 1 PE, 2 SM, 2 LPC and 5 PC levels were affected by rosuvastatin therapy (G1 vs G2), 2 PC and only 1 PE levels were decreased only by simvastatin + ezetimibe treatment (G3 vs G4). Both treatments promoted changes in the concentration levels of 5 FA, 3 LPC, 5 PE, 7PC and 6 SM. Figure 5a shows that, LPC 20:4, PE 16:0_18:2, LPC 17:0, LPC 16:0, LPC 18:1, SM 18:1;O2/20:0, presented the largest alterations by the rosuvastatin treatment. The largest alterations promoted by the simvastatin plus ezetimibe treatment were for PE 16:0_18:2, LPC 16:1 and LPC 20:4.

## Discussion

This study revealed differences in many lipid concentrations in subjects with STEMI despite comparable classic lipid profiles. After highly effective lipid-lowering therapies (rosuvastatin or simvastatin + ezetimibe), similar trend in the lipid composition were observed for both therapies. However, differences were noted between the lipid-lowering therapies for many lipids such as FA, LPC, PC, PE, CE, Cer and SM and these findings seem relevant, due to the involvement of these lipid classes in crucial mechanisms of atherosclerosis ^12–17^. These changes were more pronounced by rosuvastatin therapy, and the significant decrease in Cer observed only with this lipid-lowering drug seems important, as Cer has been considered a robust predictor for cardiovascular events, independently of LDL-C as well as for cardiovascular mortality^18,19^. It should be pointed out that, although in the lipid class evaluation, the sum of individual lipids from the same class presented significant changes only for SM class with simvastatin plus ezetimibe therapy, the statistical analysis of individual lipid species has not shown that the treatment altered lipids from many other classes (FA, PE, LPC, PC). These findings highlight the importance of evaluating lipid species individually. Lipids with different fatty acid chains, from the same class, may present opposite effects in CVD risk. In fact, lipidomic studies have shown that PC containing polyunsaturated fatty acyl chains were negatively associated with CVD risk, while saturated and monounsaturated fatty acyl chains were deleterious for CVD risk^20^.

Inflammation and oxidative stress have been considered as a part of the pathophysiology of acute coronary syndromes and their recurrences^13,21–23^. Although FA and their esters constitute the major sources of energy for the heart muscle, their excess has profound effects on the heart causing an enhanced susceptibility to inflammation, oxidative stress and ischemic damage (fibrosis and hypertrophy)^24^. Different FA can be metabolized into pro- and anti-inflammatory signalling molecules. In particular, some n-6 FA (first and foremost arachidonic acid) are precursors to pro-inflammatory molecules (primarily prostaglandins)^24^, while saturated fatty acids (SAFAs) act as major inducers of inflammation through several mechanisms, such as the activation of toll-like receptor-4 (TLR4), which promotes the production of inflammatory cytokines (IL-1beta; IL-6)^25^. Endothelial dysfunction due to the NF-kappa B activation is also induced by SAFA, resulting in increased superoxide production, while NLRP3 inflammation activation increases endothelial permeability^26,27^. In our study, in the two arms of therapy, lower concentrations of FA (SAFAs, monounsaturated fatty acids—MUFAs and polyunsaturated fatty acids—PUFAs) were observed after 1 month of treatment. These results suggest a potential beneficial effect of therapies on CVD. Interesting to note, both therapies promoted similar reduction in FA, but for FA 16:0, it was significantly reduced at D30 only in the rosuvastatin arm. Our results are in line with a meta-analysis of clinical studies involving atorvastatin and simvastatin published by Sahebkar et al. showing reduction in free fatty acids levels independently of treatment duration, dose and magnitude of reduction in LDL-C levels^28^.

LPC is increasingly recognized as a key factor positively associated with atherosclerosis development and cardiovascular diseases^29,30^. However, findings from recent clinical studies have suggested potential biomarkers of positive prognostic making those studies controversial^20^. A key issue is the complexity of the enzyme cascade involved in LPC metabolism, which shows that a long way must be traveled until the understanding of the results reported in literature^31^.

In our study, LPCs were significantly increased with rosuvastatin treatment at D30, of which five were responsible for this increase (LPC 15:0, LPC 16:1, LPC 17:0, LPC 18:1 and LPC 20:4), while only three of them had their levels altered after simvastatin + ezetimibe at D30 (Table S3). Interestingly, in agreement with our findings, LPC 18:1 and LPC 20:4 has shown a negative association with cardiovascular events in three different lipidomic studies, as reported by Ding et al*.*. Fernandez, et al.studying plasma lipid composition and risk of developing cardiovascular disease also observed a decrease in LPCs associated with CVD^20,32^. Other interesting study on lipid profile of rosuvastatin in humans has also highlighted changes in LPC and PC compound classes, suggesting an effect of rosuvastatin in LPC and PC metabolism^10^. Although no significant differences between therapies were observed in the D1 (G1 vs. G3) and D30 (G2 vs. G4) for LPC compounds class, temporal % changes in the rosuvastatin arm were higher than simvastatin/ezetimibe.

Some lipid composed of odd chain fatty acids were significantly altered by both treatments. Aforetime, odd chain fatty acids were associated solely with diet, however nowadays they are recognized as products of catabolism of branched-chain amino acids. Branched-chain amino acids were positively associated with CVD risk and type 2 diabetes^33^. Furthermore, Khaw et al. have associated odd chain phospholipids with lower cardiovascular risk^34^. Also, in the study of Ward-Caviness et al. a standard deviation increase in log-transformed concentration of LPC 17:0 negatively correlated with the risk of incident myocardial infarction in three cohorts^35^. Interestingly, our study shows that rosuvastatin therapy increased LPC 17:0 in more than 50% at D30, which could contribute to a cardiovascular protective effect.

PE and PC were reduced after treatment with both lipid-lowering therapies. High levels were found in rosuvastatin group for most of the compounds.

In addition, our study showed that both lipid-lowering therapies decreased SM concentrations at D30. Sphingomyelin was measured in the large Multi-Ethnic Study of Atherosclerosis (MESA), and the authors reported a modest negative association with incident CVDs, after adjustment for lipoproteins and full adjustment for other risk factors^36^. Higher decrease with rosuvastatin compared with atorvastatin treatments was found in the SM/SM + PC ratio^37^. The same was found here, for rosuvastatin and simvastatin + ezetimibe comparison. These results corroborate Choi et al*.* findings, that reported a significant decrease in SM, TG and PE, especially in PE 18:0_18:2 levels, and an increase in LPC 20:4 and LPC 18:1^10^. In 2018, Lee et al*.* hypothesized that these increases may be due to pleiotropic effects of statins, since phospholipase A2, which is the main enzyme involved in LPC metabolism, is inhibited by rosuvastatin^38^.

Besides the superiority in changing lipid concentration for rosuvastatin in the altered classes compared to simvastatin + ezetimibe, only rosuvastatin promoted significant reduction in CE and Cer compounds.

Ng et al*.* studying the dose-dependent effects on plasma sphingolipidome and phospholipidome in the metabolic syndrome found that rosuvastatin at both 10 and 40 mg/d significantly reduced the concentrations of total and individual plasma sphingolipids and phospholipids with evidence of dose-dependent effects^39^. For CE, as the results found for LPC, controversial findings have been reported, but Stegemann et al. applying mass spectrometry-based lipidomic profiling, reported an association of CE with cardiovascular disease over a 10-year observation period^40^.

Considering the correlations between lipids and classic clinical parameters, it is interesting to note the number of correlations found at G1 compared to other groups (G2, G3, and G4). The lack of correlations for those groups indicates that lipidomic measurements and classic lipid profile addresses different information. In this work, PC, SM and TG were positively correlated to LDL-C for both lipid-lowering therapies at D1, but a negative or no correlation between HDL-C and TG was found, mainly when rosuvastatin was administrated at D30. There is an expected inverse association between HDL-C and TG in diabetic and overweight patients. Patients with insulin resistance have delayed hydrolysis of TG-rich lipoproteins due to reduced lipoprotein lipase activity. As a result, fewer phospholipids and surface components are transferred to HDL, decreasing its effectiveness for the reverse cholesterol transport. Therefore, lower concentrations of cholesterol from HDL are found.

A previous study reported that LPC are predominantly found in HDL, Cer in LDL, and PC are present in both lipoproteins (HDL and LDL)^20^. Studies examining CVDs with lipidomics found that after adjusting for HDL-C and LDL-C levels, only PC remained associated with cardiovascular events^20,33,41–45^.

In this work, LDL-C was reduced with both lipid-lowering therapies at similar % changes. Otherwise, Cer was reduced significantly, only with rosuvastatin at D30. As a reduction of LDL-C and Cer is expected to contribute to lower residual cardiovascular risk, rosuvastatin presented better results than simvastatin + ezetimibe. Considering classic lipid parameters, rosuvastatin treatment decreased in 17% HDL-C level after one month. Another study showed that patients on rosuvastatin therapy may have either increased or decreased the HDL-c after 4 weeks of treatment. However, these changes in HDL-C did not affect the incidence of major cardiovascular events in one year follow-up^46^.

In summary, regarding to the changes in the classic lipid profile, our results are in agreement with previous studies^38,47–50^. Interestingly, the sum of our findings corroborates with a review article recently published by Rai & Bhatnagar showing decreased LPC 16:0 in hyperlipidemia causative disorders, such as high-fat diet, obesity and diabetes. LPC decrease was accompanied by increase in FA and Cer. The authors also highlighted the association of hyperlipidemia with an increase in small-chain fatty acids, SAFA content of DG, TG and PC lipid classes, factors associated with CVD risk^51^. No direct comparisons between rosuvastatin and simvastatin + ezetimibe in cardiovascular outcomes have been reported.

This study compared two highly effective lipid-lowering therapies in the acute phase of myocardial infarction. However, we are unable to estimate the effects of the acute myocardial infarction per se on lipid composition. It is expected some decrease in lipids due to the healing process involving the necrotic and ischemic myocardium, but these changes have been reported as insignificant in the following days after the acute coronary event^50^. In addition, the myocardial injury estimated by troponins was similar in both arms of lipid-lowering treatment. Our sample size is relatively small, but the patients included in the study were all submitted to same treatment strategy (pharmacoinvasive) with similar characteristics at baseline.

## Conclusions

In spite of comparable classic lipid profile at baseline and after the exposure to treatments, significant differences in lipid composition were found between the two highly effective lipid-lowering therapies. To note, higher % changes in the rosuvastatin arm of therapy compared to simvastatin + ezetimibe were identified, and significant changes for CE and Cer were observed only in the rosuvastatin group. Regarding the correlation studies, we found that lipidomic analysis and classic clinical exams account for different information in both lipid-lowering therapies. In summary, our results indicate important differences in lipid composition that cannot be identified by the classic lipid profile between the studied lipid-lowering therapies. These differences may account for residual cardiovascular risk.

## Methods

### Reagents and standards

Methanol, 1-propanol and dichloromethane in HPLC grade were purchased from JTBAKER (Avantor Performance Materials, Mexico, Mexico). Water was purified by the Milli Q system (Millipore Waters, Darmstadt, Germany). Ammonium acetate was obtained from Sigma-Aldrich (Saint Louis, MO, USA). The Lipidyzer isotope labeled internal standards mixture kit consisting of 54 isotopes from 13 lipid classes (LPC, lysophosphatidylethanolamines (LPE), PC, PE, Sphingomyelin (SM), diacylglycerols (DG), TG, FA, Cholesterol ester (CE), Ceramide (Cer); dihydroceramides (DhCer), hexosylceramides (HexCer), lactosylceramides (LacCer) was purchased from Sciex (Framingham, MA, USA).

### Study design

This prospective, randomized, open label study was delineated to evaluate differences in the composition of lipids in patients with STEMI at D1 and at D30 after implementing the two lipid-lowering therapies (rosuvastatin 20 mg [Crestor, AstraZeneca] or simvastatin 40 mg combined with ezetimibe10 mg [Vytorin, MSD]). Plasma of patients were categorized in four groups: patients in the rosuvastatin group at D1 (G1) and at D30 (G2); patients treated with simvastatin + ezetimibe at D1 (G3) and at D30 (G4). These two lipid-lowering therapies were chosen to promote similar changes in the classic lipid profile, allowing the comparison of the more effective inhibition of cholesterol synthesis (rosuvastatin) with the combined mechanisms of LDL-C lowering (inhibition of cholesterol synthesis and inhibition of intestinal cholesterol absorption by simvastatin/ezetimibe). Patients were randomized 1:1 for the lipid-lowering therapy using a central computerized system (battle-ami.huhsp.org.br). All patients followed similar protocol, receiving dual antiplatelet therapies, betablockers, and renin-angiotensin system blockers and they were referred to coronary angiogram, and percutaneous coronary intervention, when needed, in the first 24 h of STEMI.

### Cohort

The study included mainly middle aged males, approximately half of them with type 2 diabetes. From the 25 patients consecutively screened for the trial, three were not eligible due to inclusion/exclusion criteria and two did not complete trial (one patient died in the first month e other was hospitalized due to heart failure). Table 1 shows the main characteristics of study population. The cohort is part of the B And T Types of Lymphocytes Evaluation in Acute Myocardial Infarction (BATTLE-AMI) study (NCT02428374)^52^. They had no prior MI and were naive for lipid-lowering treatment. All patients were submitted to pharmacological thrombolysis with tenecteplase in the first 6 h of STEMI, followed by coronary angiogram and percutaneous intervention when needed in the first 24 h of STEMI (pharmacoinvasive strategy). Key exclusion criteria included hemodynamic instability, autoimmune disease, known malignancy, pregnancy and signs of active infections. All patients received the study medications from the hospitalization as well as at hospital discharge. These patients were monitored by phone and followed up in our outpatient clinic (Hospital Sao Paulo—UNIFESP). At each visit, the patients brought the boxes of their medications to check their adherence.

**Table 1:** Characteristics of the study population and classic lipid profile at baseline and after treatment.

Parameters	Rosuvastatin (n = 10)	Simvastatin/ezetimibe (n = 10)	*p*-value
Age, years, median (IQR)	62 (59–64)	53 (48–62)	0.06
Male gender, n (%)	7 (70)	8 (80)	0.61
Weight, kg	76.2 ± 11.7	74.5 ± 10.9	0.78
BMI, kg m^−2^, median (IQR)	25.3 (24.4–29.7)	29.1 (28.5–32.3	0.32
Diabetes, n (%)	4 (40)	6 (60)	0.37
Hypertensives, n (%)	7 (70)	7 (70)	1.00
**Baseline**			
HbA1c, %	6.2 ± 1.6	6.9 ± 1.7	0.83
Glucose, mg dL^−1^	155 ± 61	171 ± 78	0.41
Creatinine, mg dL^−1^	0.96 ± 0.21	0.95 ± 0.20	0.85
GFR, mL min^−1^ m^2^	77 ± 15	86 ± 21	0.32
Troponin T, ng L^−1^	5713 ± 3366	7770 ± 7053	0.42
Cholesterol, mg dL^−1^	202 ± 44	204 ± 47	0.91
LDL-cholesterol, mg dL^−1^	131 ± 29	134 ± 39	0.85
HDL-cholesterol, mg dL^−1^	41 ± 9	39 ± 13	0.71
Triglycerides, mg dL^−1^	171 ± 106	189 ± 126	0.75
**After 30 days**			
Cholesterol, mg dL^−1^	106 ± 13	118 ± 24	0.18
LDL-cholesterol, mg dL^−1^	51 ± 11	59 ± 21	0.30
HDL-cholesterol, mg dL^−1^	34 ± 3	35 ± 6	0.60
Triglycerides, mg dL^−1^	123 ± 33	152 ± 66	0.23

Data are mean ± SD unless otherwise stated. Comparisons were examined by unpaired Student´s t test or by the non-parametric Mann–Whitney U test. Categorical variables were tested by Pearson’s Chi-square test. *BMI* body mass index, *HbA1c* glycated hemoglobin, *GFR* glomerular filtration rate.

### Clinical measurements

Fasting blood samples were collected, in the morning at D1 and at D30 after lipid-lowering therapy, in tubes containing EDTA, followed by centrifugation at 1300*g* for 15 min, at room temperature and storage at − 80 °C before analysis. All samples for general biochemical tests, including the classic lipid profile were performed in the Central Laboratory of the University Hospital and the LDL-C was estimated by the Friedewald equation. Biochemical determination of TC, HDL-c and TG* was performed by enzymatic colorimetric assays with commercial kits from Roche in Cobas C 501 module.

### Lipid extraction

Lipid extraction was carried out by a modified Blight-Dyer protocol as described elsewhere^53^. Briefly, 100 μL of plasma were transferred to a borosilicate glass culture tube (16 × 100 mm)^53^. Next, 900 μL water, 2 mL methanol, and 900 μL dichloromethane were added to all samples and the mixture was vortexed for 5 s. Samples were left to incubate at room temperature for 30 min. Next, another 1 mL water and 900 μL dichloromethane were added to the tube, followed by gentle vortexing for 5 s, and centrifugation at 2500*g* at 15 °C for 10 min. The bottom organic layer was transferred to a new tube and 1.8 mL dichloromethane were added to the original tube for a second extraction. The combined extracts were concentrated under nitrogen. Exactly 100 μL of the isotope labeled internal standards mixture were added to the dried extract and another 30 min incubation was allowed until equilibrium is reached. Finally, 250 μL mobile phase solution (10 mmol L^−1^ ammonium acetate in 50:50 methanol:dichloromethane) were added. IS mixture is composed by 54 labeled lipid species that covers 10 main lipid classes found in human plasma with different final concentrations reflecting their physiological concentrations^54^.

### Lipid quantification

Quantitative lipidomics was performed with the Sciex Lipidyzer platform configured by an ExionLC AD instrument (Sciex) coupled to a QTRAP 5500 mass spectrometer (Sciex) equipped with SelexION for differential mobility spectrometry (DMS) and electrospray ionization (ESI) source. The solvent 1-propanol was used as the chemical modifier for the DMS. Samples were introduced to the mass spectrometer by flow injection at 8 µL min^−1^. Each sample was injected twice, with the DMS on (PC/PE/LPC/LPE/SM) and off (CE/Cer/DG/FA/TG). Over 1100 lipid species and 54 labeled internal standards were monitored by selected reaction monitoring (SRM) in positive/negative polarity switching. Positive ion mode was used to detect the lipid classes SM/DG/CE/Cer/TG and the negative ion mode to detect the lipid classes LPE/LPC/PC/PE/FA. Lipid annotation is achieved by measuring specific SRM transitions, where the monitored fragment is related to the fatty acid composition. Also, lipid class is determined by ramping the compensation voltages in the differential mobility unit of the Lipidyzer platform. DMS parameters used were: temperature = low; separation voltage = 3.5 kV and differential mobility spectrometric resolution = low. Electrospray ion source parameters were as follow: voltage (ESI +): 4.1 kV, voltage (ESI -) = − 2.5 kV, curtain gas = 17, CAD gas = Medium, Temperature = 200 °C, Nebulizing gas = 17 and heater gas − 25.

All data obtained from the Lipidyzer Platform were automatically processed in the Lipidomics Workflow Manager (LWM). Signals of all lipids obtained for each sample were quantified using the intensity of internal standard applying the Lipidyzer platform. The software calculates concentration as average intensity of the analyte MRM/average intensity of the most structurally similar IS MRM multiplied by its concentration in nmol/mL. Lipidyzer platform allowed for automated data acquisition, data processing, and reporting. A detailed description of the method can be found in previous studies^55,56,56–59^. Quality control (QC) consisted of a standard plasma sample obtained from the Lipydizer kit. The reconstituted lyophilized plasma was extracted following the procedure described previously. The QC sample was injected five times at the beginning of the randomized sample batch, every 10 injections and, at the end of the sample batch.

### Data treatment

Box-plot was performed using Minitab 17.0 (Minitab Statistical Software; https://www.minitab.com/pt-br/) and Microsoft Excel 2013. Univariate analysis (Repeated Measures ANOVA) was performed in MATLAB (The MathWorks, Inc., Natick, Massachusetts, United States) using an in-house script.

Multivariate analysis was performed in SIMCA 16 (Statistical Software Package, Umetrics, Sweden; http://umetrics.com/product/simca). Correlation graphics were performed in R 3.6.3 (The R Project for Statistical Computing; https://www.r-project.org/packages: corrplot, Hmisc, RColorBrewer, Cairo). From the original generated table compiling the lipids identified (Supplementary Table S7), only the ones that had presented concentrations (µmol L-1) in at least 80% of the samples of one group were used for data treatment. Discriminant variables were obtained not only by multivariate analysis (VIP > 1), but also by univariate evaluation by Post Hoc test with Bonferroni correction for multiple comparisons of the Repeated Measures ANOVA, as shown in Supplementary Table S3.

Data quality was carried out by inspecting the repeatability of lipids in the QC plasma sample, analyzed throughout data acquisition. More than 80% of the quantified metabolites in the QCs have acceptable coefficient of variation percentages (% CV) for peak areas; in this work, < 20% CV was used as a criterion to retain that particular component in the dataset for further evaluation, which was in agreement with the recomendation^60,61^.

### Ethics declarations

The study protocol was approved by the local ethics committee (UNIFESP IRB 0297/2014; CAAE: 71652417.3.0000.5505), which follows the Declaration of Helsinki, and written informed consent was provided by all subjects before their inclusion in the study.

## Supplementary Information

Supplementary Information.

## Data Availability

We statement that the all data generated or analyzed during this study are included in this published article and in its Supplementary Information files.

## Abbreviations

LDL-C: Low-density lipoprotein cholesterol
CVD: Cardiovascular disease
CHD: Coronary heart disease
FA: Fatty acids
NLRP3: NOD-, LRR- and pyrin domain-containing protein 3
SM: Sphingomyelin
TG*: Triglycerides
TG: Triacylglycerides
PI: Phosphatidylinositol
PE: Phosphatidylethanolamines
LPC: Lysophosphatidylcholines
PC: Phosphatidylcholines
HDL-C: High-density lipoprotein cholesterol
STEMI: ST-segment elevation myocardial infarction
TC: Total cholesterol
CE: Cholesterol ester
Cer: Ceramides
LPE: Lysophosphatidylethanolamine
PE: Phosphatidylethanolamine
SRM: Selected reaction monitoring
DMS: Differential mobility spectrometry
DhCer: Dihydroceramides
HexCer: Hexosylceramides
LacCer: Lactosylceramides
SAFAs: Saturated fatty acids
TLR4: Toll-like receptor-4
MUFAs: Monounsaturated fatty acids
PUFAs: Polyunsaturated fatty acids
OPLS-DA: Orthogonal projection to latent structures discriminant analysis
VIP: Variable importance for the projection
